# rLj-RGD3 Suppresses the Growth of HeyA8 Cells in Nude Mice

**DOI:** 10.3390/molecules22122234

**Published:** 2017-12-15

**Authors:** Yuanyuan Zheng, Li Lv, Longda Yi, Rui Wu, Rong Xiao, Jihong Wang

**Affiliations:** 1 School of Life Sciences, Liaoning Normal University, Dalian 116081, China; bio_yy_zheng@163.com (Y.Z.); Rossoneri_yld@163.com (L.Y.); m18742501231@163.com (R.W.); 2 Department of Pharmacology, Dalian Medical University, Dalian 116044, China; lv_li@126.com

**Keywords:** rLj-RGD3, HeyA8 cells, nude mice, PI3K, AKT

## Abstract

In the previous study, rLj-RGD3, a recombinant toxin protein which contains three RGD motifs, was reported to not only inhibit the proliferation of an ovarian cancer cell line, HeyA8 cells, by inducing apoptosis, but also block their adhesion, migration and invasion processes. However, whether rLj-RGD3 could also suppress the tumor growth in HeyA8 xenografted mice has not been reported yet. In the present study, rLj-RGD3 was intraperitoneally injected in the nude mice bearing HeyA8 tumors. Compared with the control group (normal saline), rLj-RGD3 inhibited the tumor growth significantly in the HeyA8 xenografted mice in a dose-dependent manner without affecting their body weights. Based on the H&E, Hoechst 33258 and TUNEL staining assays, as well as western blot analysis, rLj-RGD3 reduced the weight and volume of the solid tumors, probably by disturbing the tissue structure, inducing apoptosis and suppressing the FAK/PI3K/AKT pathway. Most importantly, rLj-RGD3 was found to prolong the survival days of the ovarian tumor xenografted mice, which suggested rLj-RGD3 might act as an effective and safe drug to treat ovarian cancer patients.

## 1. Introduction

To date, ovarian cancer is also one of the most important cancers which causes the death of women all around the world [1]. Based on their characteristics, including histotype, pathogenesis, tumor growth, prognosis and response to therapy, ovarian cancers are mainly divided into two groups: low-grade and high-grade [2]. Regrettably, approximately 70% of the ovarian cancers are diagnosed at high grade, which results in the high mortality in women [2]. At present, lots of hypotheses have been suggested, however, the exact pathogenesis is still not well understood [3]. Usually, even after treatment with cytoreductive surgery and adjuvant chemotherapy, ovarian cancer patients might still face a high recurrence rate [1,4]. Thus, a great number of studies have paid attention to the pathogenesis of ovarian cancer and the screening of novel drugs in order to treat ovarian cancer patients effectively.

Integrins are a group of receptors located on the cell surface that can mediate the attachment of cells to the extracellular matrix (ECM) [5]. As integrins are closely related to the progression of tumors, clinical studies usually consider integrins as ideal targets for the treatment of cancer [5]. Among the integrins, α5 integrin was reported to promote the attachment and invasion of RMUG-S cells which are known ovarian tumor cells [6]. Based on western blot analysis, the expression of α5 integrin was also detected in seven other ovarian cancer cell lines, including SKOV-3ip1, A2780, HeyA8, OVMZ-6, RMUG-L, CAOV-3, and OVCAR-5, suggested that antagonists which target α5 integrin might act as effective anti-tumor drugs to treat ovarian cancer [6]. Furthermore, other integrins including α6β2, αvβ3, α4β1-integrin were also proved to participate in the progression of ovarian cancers [7].

rLj-RGD3 is a recombinant protein which was firstly identified in the buccal glands of the *Lampetra japonica* (*L. japonica*) [8]. Our previous studies have shown that rLj-RGD3 was able to suppress the proliferation of Hela cells, ECV304 cells, MCF-7/Adr cells, MCF-7 cells, 786-0 cells, Panc-1 cells, as well as HeyA8 cells [8,9,10,11,12,13]. In 2017, rLj-RGD3 expressed without the His-tags was reported to suppress the activity of HeyA8 cells in vitro experiments [13]. Regrettably, the authors did not study the effects of rLj-RGD3 on the HeyA8 cells in vivo. In the present study, nude mice bearing HeyA8 xenografts were obtained and the effects of rLj-RGD3 on the length of survival, body weight, tumor growth, tumor volume, and tumor weight of the above nude mice were determined. Furthermore, the related mechanism and signal pathway were also discussed.

## 2. Results

### 2.1. rLj-RGD3 Suppressed the Tumor Growth in HeyA8 Xenografted Mice without Affecting Their Body Weight

**Figure 1:**
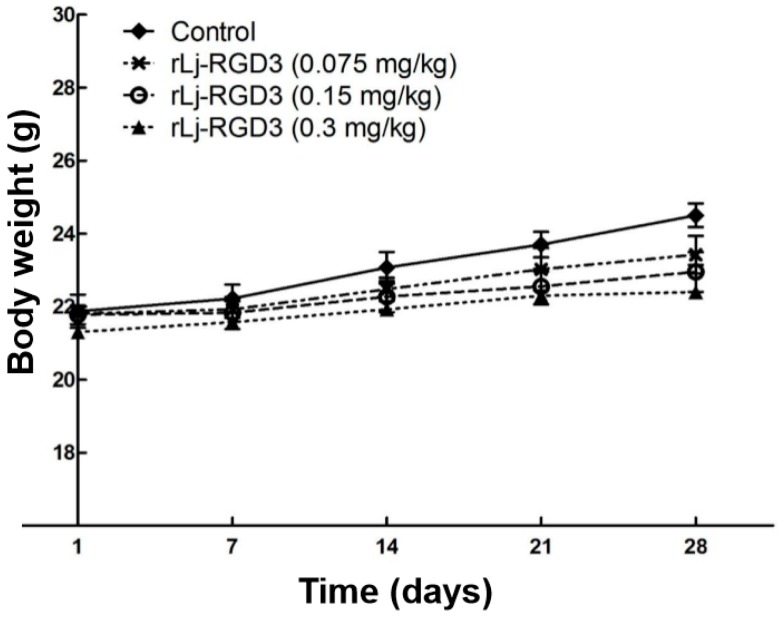
rLj-RGD3 did not affect the body weight of HeyA8 xenografted mice significantly (*n* = 8).

**Figure 2:**
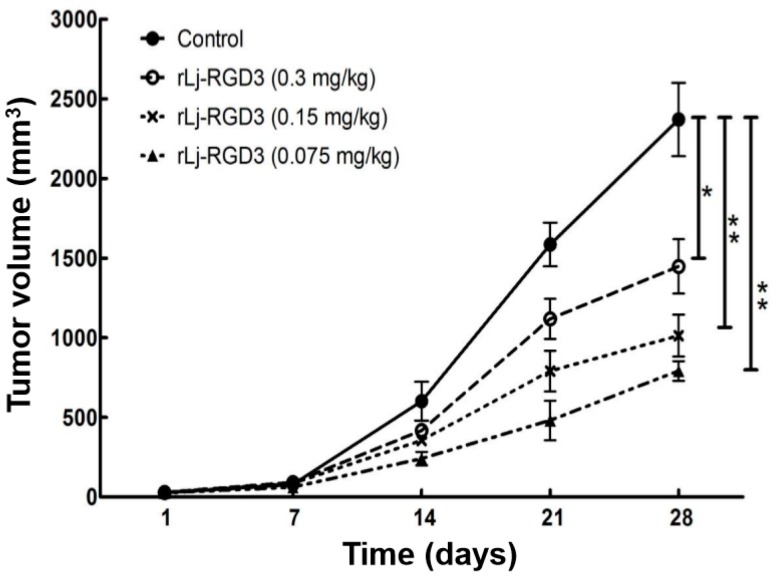
rLj-RGD3 inhibited the growth of the tumors in HeyA8 xenografted mice (*n* = 8). Relative to the control group: * *p* < 0.05; ** *p* < 0.01.

**Figure 3:**
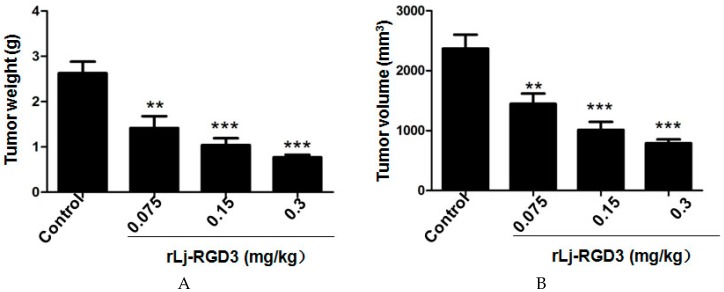
rLj-RGD3 suppressed the weight (**A**) and volume (**B**) of the solid tumors in HeyA8 xenografted mice (*n* = 8). Relative to the control group: ** *p* < 0.01; *** *p* < 0.001.

Previous studies have shown that compounds or proteins such as doxorubicin, which could suppress the growth and metastasis of the tumors in vivo, also reduced the body weight of the xenografted mice [14]. Thus, we first detected whether rLj-RGD3 could also affect the body weight of the HeyA8 xenografted mice. As shown in Figure 1, rLj-RGD3 did not affect the body weight of the HeyA8 tumor-bearing mice significantly when compared with the control (normal saline) group. As shown in Figure 2, the tumor volume increased significantly as the time went on. Compared with the normal saline group, rLj-RGD3 suppressed the increase of the tumor volume in HeyA8 xenografted mice in a dose-dependent manner (Figure 2). After 28 days, both the volume and weight of the excised tumors were obviously smaller and lighter in the rLj-RGD3 administrated groups than that in the normal saline group (Figure 3). The average tumor volume in the normal saline, 0.075 mg/kg, 0.15 mg/kg and 0.3 mg/kg rLj-RGD3 groups was 2370.3 ± 230.8 mm^3^, 1447.8 ± 171.0 mm^3^ (*p* < 0.01), 1013.5 ± 131.9 mm^3^ (*p* < 0.001) and 791.8 ± 61.5 mm^3^ (*p* < 0.001), respectively. The inhibitory rate of rLj-RGD3 on the tumor volume was 40.0%, 57.2% and 66.6%, respectively. The average tumor weight in the normal saline, 0.075 mg/kg, 0.15 mg/kg and 0.3 mg/kg rLj-RGD3 groups was 2.62 ± 0.25 g, 1.42 ± 0.26 g (*p* < 0.01), 1.04 ± 0.15 g (*p* < 0.001) and 0.77 ± 0.06 g (*p* < 0.001), respectively. The inhibitory rate of rLj-RGD3 on the tumor weight was 45.8%, 61.5% and 70.6%, respectively.

### 2.2. rLj-RGD3 Improved the Survival Days in the HeyA8 Xenografted Mice

**Figure 4:**
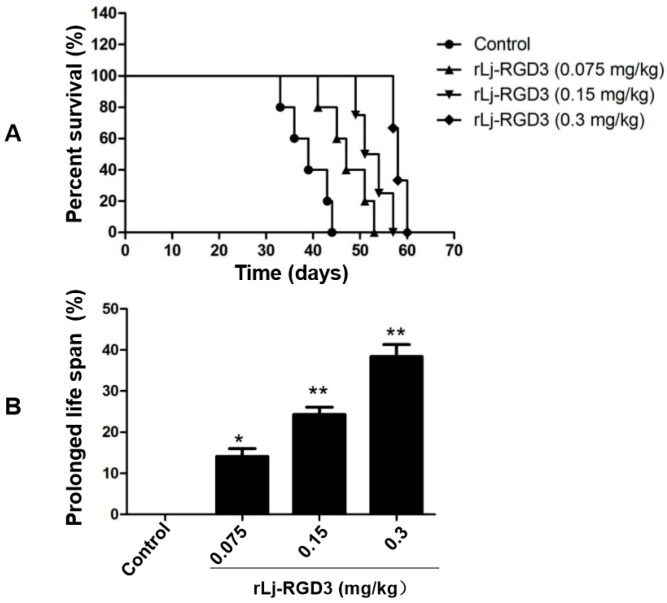
rLj-RGD3 prolonged the survival days in HeyA8 xenografted mice (*n* = 7). The survival plot for tumor-bearing mice treated with normal saline or rLj-RGD3 was shown in panel A. The prolonged life span was shown in panel B. Relative to the control group: * *p* < 0.05; ** *p* < 0.01.

As rLj-RGD3 significantly suppressed the tumor growth in HeyA8 xenografted mice, the question was whether rLj-RGD3 would improve the survival days of these mice. As shown in Figure 4, the average survival days of these HeyA8 xenografted mice in the normal saline, 0.075 mg/kg, 0.15 mg/kg and 0.3 mg/kg rLj-RGD3 treating groups were 41.4 ± 1.2, 47.9 ± 3.0 (*p* < 0.05), 52.2 ± 2.6 (*p* < 0.01), and 58.5 ± 1.3 (*p* < 0.01) days, respectively. Compared with the normal saline group, the prolongation rate on the survival of the HeyA8 xenografted mice in the 0.075 mg/kg, 0.15 mg/kg and 0.3 mg/kg rLj-RGD3 groups was 16.0% (*p* < 0.05), 26.1% (*p* < 0.01) and 41.3% (*p* < 0.01), respectively (Figure 4). This indicated that rLj-RGD3 increased the survival days of HeyA8 xenografted mice in a dose-dependent manner.

### 2.3. rLj-RGD3 Changed the Histological Characteristics and Induced Apoptosis in the Tumors of HeyA8 Xenografted Mice

**Figure 5:**
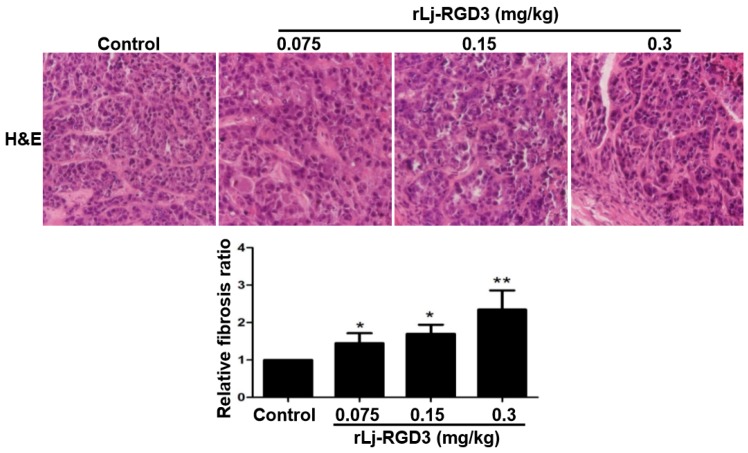
rLj-RGD3 changed the histological characteristics (400×) in the tumors of the HeyA8 xenografted mice. After treating rLj-RGD3, more gaps and fibrosis were observed in the H&E staining assay. The relative fibrosis ratio was calculated based on the formula mentioned in the methods and shown in a histogram. Relative to the control group: * *p* < 0.05; ** *p* < 0.01.

In order to further clarify the anti-tumor effects of rLj-RGD3 on HeyA8 xenografted mice, a hematoxylin and eosin (H&E) staining assay was used to observe the tumor tissues after the administration of rLj-RGD3. As shown in Figure 5, the HeyA8 cells in the solid tumor tissues arranged more closely in the normal saline group than that in the rLj-RGD3 groups. After treating with rLj-RGD3, more gaps were observed. The density of the HeyA8 cells also decreased significantly in the rLj-RGD3 treating groups when compared with the normal saline group (Figure 5). Furthermore, fibrosis was also observed in the tumor tissues treated with rLj-RGD3 (Figure 5). As shown in Figure 5, the fibrosis ratio in the 0.075 mg/kg, 0.15 mg/kg and 0.3 mg/kg rLj-RGD3 groups was 1.4 fold (*p* < 0.05), 1.7 fold (*p* < 0.05), and 2.3 fold (*p* < 0.01) of the control group, respectively.

**Figure 6:**
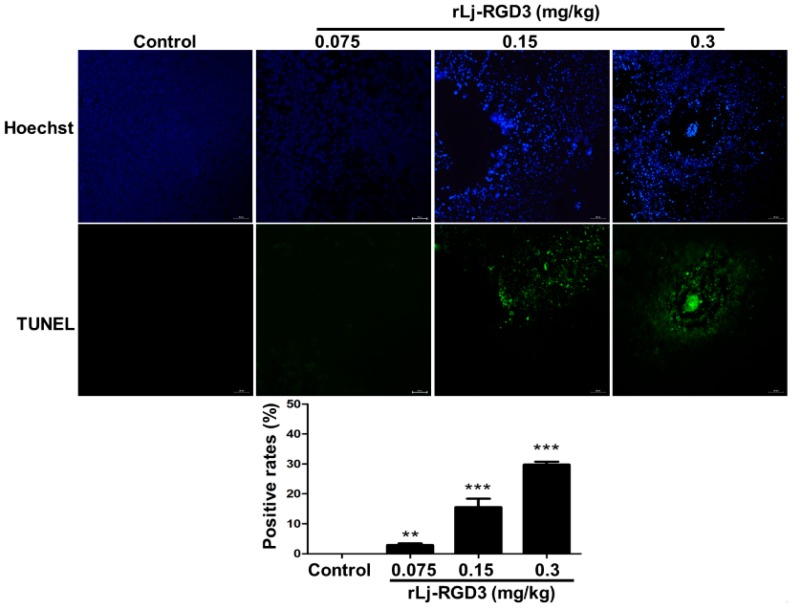
rLj-RGD3 induced apoptosis (200×) in the tumors of the HeyA8 xenografted mice. The nuclei (blue signals) of the HeyA8 cells in the solid tumor tissues are shown in the first line. The green signals which indicated the apoptotic cells in the TUNEL assay are shown in the second line. The positive rates of the apoptotic cells were shown in a histogram. Relative to the control group: ** *p* < 0.01; *** *p* < 0.001.

**Figure 7:**
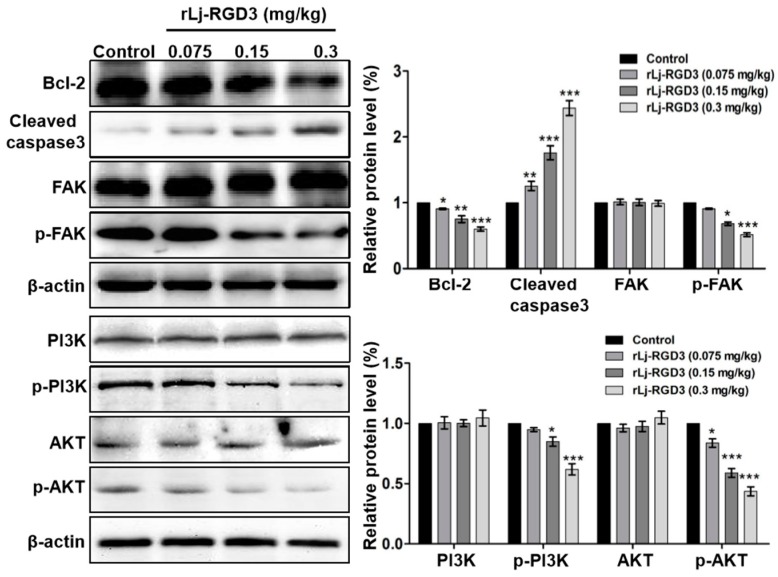
rLj-RGD3 changed the level of Bcl-2, cleaved caspase 3, p-FAK, p-PI3K and p-AKT in the tumors of nude mice bearing HeyA8 xenografts. β-actin was used as a control. Relative to the control group: * *p* < 0.05; ** *p* < 0.01; *** *p* < 0.001.

As rLj-RGD3 induced morphological changes, classic apoptotic assays were also performed to assess whether rLj-RGD3 could induce apoptosis in vivo. As shown in Figure 6, Hoechst 33258 staining assay showed the blue signals in the rLj-RGD3 groups were brighter than that in the normal saline group. Similarly, a TUNEL assay also showed that rLj-RGD3 increased the number of the apoptotic cells in the tumor tissues of the HeyA8 xenografted mice (Figure 6). The positive rates of the apoptotic cells in the 0.075 mg/kg, 0.15 mg/kg and 0.3 mg/kg rLj-RGD3 treatment groups were 2.8% (*p* < 0.01), 15.6% (*p* < 0.001), and 32.1% (*p* < 0.001), respectively (Figure 6). A western blot assay showed that rLj-RGD3 reduced the level of Bcl-2, but increased the level of cleaved caspase 3 in the tumor tissues of the HeyA8 xenografted mice (Figure 7). Compared with the normal saline group, 0.075 mg/kg, 0.15 mg/kg and 0.3 mg/kg rLj-RGD3 reduced the level of Bcl-2 by 16.2% (*p* < 0.05), 24.9% (*p* < 0.01) and 40.0% (*p* < 0.001), while increased the level of cleaved caspase 3 by 25.5% (*p* < 0.01), 76.0% (*p* < 0.001) and 144.0% (*p* < 0.001), respectively. The above indicated that rLj-RGD3 induced apoptosis in vivo.

### 2.4. rLj-RGD3 Suppressed the FAK/PI3K/AKT Pathway in the Tumors of HeyA8 Xenografted Mice

In our in vitro studies, rLj-RGD3 decreased the level of p-focal adhesion kinase (FAK) in HeyA8 cells. Similarly, rLj-RGD3 was also able to reduce the level of p-FAK in the tumor tissues of HeyA8 xenografted mice (Figure 7). Compared with the normal saline group, 0.15 mg/kg and 0.3 mg/kg rLj-RGD3 reduced the level of p-FAK by 31.8% (*p* < 0.05) and 48.5% (*p* < 0.001), respectively. Furthermore, rLj-RGD3 inhibited the activation of phosphoinositide 3-kinase (PI3K)/protein kinase B (AKT) pathway in vivo. As shown in Figure 7, rLj-RGD3 reduced the level of p-PI3K and p-AKT in the solid tumor tissues. Compared with the normal saline group, the inhibitory rate on the level of p-PI3K in the tumors treated with 0.075 mg/kg, 0.15 mg/kg and 0.3 mg/kg rLj-RGD3 was 5.1%, 15.1% (*p* < 0.05) and 38.1% (*p* < 0.001), respectively, and the rate of inhibition on the p-AKT level was 16.2% (*p* < 0.05), 41.1% (*p* < 0.001) and 56.3% (*p* < 0.001), respectively (Figure 7).

## 3. Discussion

The tripeptide Arg-Gly-Asp (RGD) motif within ECM proteins including fibronectin, vitronectin, collagen IV, and laminin could recognize eight integrin dimers located at the cell surface, which finally mediates the adhesion and communication between the cells [15,16]. As transmembrane adhesion molecules, integrins can not only receive signals outside the cells, but also output the signals inside the cells [17]. Thus, integrins play very important roles in a variety of signal pathways [17]. Not surprisingly, lots of studies have also reported that integrins are closely related to the tumor progression, as the expression level of several integrins such as αvβ3, αvβ5, αvβ6, αvβ8, and α5β1 is usually up-regulated in the tumor cells [15]. More importantly, integrins are also expressed in the other cell types in the tumor microenvironment (TME) except the tumor cells, suggested that the intercellular communication in TME regulated by the integrins might further promote the metastasis of the tumor cells [17,18]. Therefore, more and more studies have tried to target special integrins to suppress the proliferation and metastasis of tumor cells. To date, antagonists and antibodies against integrins have been the main focus in clinical studies [15]. Although a cyclized pentapeptide named as cilengitide showed encouraging effects in patients with glioblastoma, adverse effects such as intracranial hemorrhage were also observed [19]. Recently, more novel integrin antagonists have also been designed and investigated to exclude the above side effects.

At present, native RGD motif-containing peptides or proteins were mostly reported in the venom of snakes or the salivary glands of ticks, flies, tabanids, mosquitoes, and leeches, and not only suppressed the tumor growth and metastasis, but also inhibit platelet aggregation [20,21,22,23,24,25]. In 2010, a novel protein which contains three RGD motifs (Lj-RGD3) was first found in the buccal glands of *L. japonica*, one of the most primitive vertebrates [8]. Like the other RGD toxins, rLj-RGD3 could also target integrins, including αvβ3, αvβ5 and β1 [8]. According to our previous study, rLj-RGD3 was recombinant and expressed without His-tags in order to meet the criterion of genetic engineering drugs [13]. This His-tags-removed rLj-RGD3 could also suppress the proliferation, adhesion, migration and invasion of HeyA8 cells in in vitro experiments, which is similar to the manner of the other RGD motif-containing proteins [13,20,23]. Regrettably, the previous study did not report whether rLj-RGD3 could also work in ovarian tumors in vivo. In the present study, the His-tags-removed rLj-RGD3 was proved to effectively suppress the growth of HeyA8 tumors in xenografted mice. This suggested rLj-RGD3 might have the potential to be used as an anti-tumor drug to treat patients with ovarian cancer. At present, some anti-tumor compounds or proteins possess cytotoxicity which might cause injuries in the normal tissues of the mice and reduce their body weight [14]. During the treatment of rLj-RGD3, the body weight of these xenografted mice did not change significantly. As shown in the previous study, rLj-RGD3 also did not affect the normal tissues including lungs, hearts, livers, spleens and kidneys of the Sprague Dawley (SD) rats, which further indicated that rLj-RGD3 might be safe in clinical studies [26]. Except for the suppression of tumor volume and weight, rLj-RGD3 was also found to prolong the duration of survival in the xenografted mice. Whether rLj-RGD3 might also improve the survival rates in clinical studies still need further investigation.

As rLj-RGD3 decreased the mitochondrial membrane potential and changed the content of proteins such as BCL2, BAX, caspase 3, and cleaved caspase 3, rLj-RGD3 induced apoptosis in HeyA8 cells in a mitochondrial-dependent pathway [13]. In fact, the present study also showed that the number of apoptotic cells in the rLj-RGD3 groups was relatively greater than that in the control group, and the level of Bcl-2, as well as cleaved caspase 3 changed, which further proved that the apoptosis in HeyA8 cells induced by rLj-RGD3 might be the major cause to result in the suppression on solid tumor growth in vivo.

Based on the previous study, rLj-RGD3 reduced the ratio of p-FAK/FAK in HeyA8 cells [13]. Usually, the RGD toxins target integrins on the tumor cell surface and then suppress the activation of its downstream molecule FAK. As we all know, FAK is able to promote the proliferation and metastasis of the tumor cells and its expression and phosphorylation level usually up-regulate during the tumor progression [27]. Usually, over-expression of FAK is closely associated with the activation of the PI3K and AKT signaling pathways which also participate in the regulation of tumor cells’ proliferation [27,28]. In the present study, rLj-RGD3 was found to suppress the activation of FAK, PI3K and AKT in the solid tumors of HeyA8 xenograted mice. This means that rLj-RGD3 might target integrins in HeyA8 cells and then suppress the activation of FAK, as well as its downstream PI3K/AKT to inhibit the growth of tumors in nude mice.

## 4. Materials and Methods

### 4.1. The Preparation of rLj-RGD3

Based on the previous study, rLj-RGD3 without His-tag was obtained through a nickel-column (GE Healthcare, Chicago, IL, USA) and run on reducing Tricine sodium dodecyl sulfate-polyacrylamide gel electrophoresis (SDS-PAGE) [13]. Subsequently, the purified rLj-RGD3 was desalted and concentrated with a lyophilizer (Christ, Osterode am Harz, Germany). Finally, Coomassie Brilliant Blue G250 was used to measure the concentration of the purified rLj-RGD3.

### 4.2. HeyA8 Cells Culture

Human ovarian cancer cell lines (HeyA8 cells) were acquired from Professor Pixu Liu (Dalian Medical University, Dalian, China). The HeyA8 cells were maintained in Dulbecco’s Modified Eagle Medium (DMEM, GIBCO, Thermo Fisher Scientific, Waltham, MA, USA) in the presence of 10% (*v*/*v*) fetal bovine serum (FBS, GIBCO, Thermo Fisher Scientific), 100 U/mL penicillin G and 100 μg/mL streptomycin in a CO_2_ incubator (Thermo Fisher Scientific) at 37 °C.

### 4.3. Animals

Sixty nude mice (30 males and 30 females) were obtained from the Dalian Medical University Laboratory Animal Centre (Permit number: SCXK2013-0003). The body weight of these nude mice was about 18–22 g. All the mice were fed with the purified rodent diet in the Animal Centre (temperature: 20–22 °C; humidity: 50–80%). And the procedures about animal studies were approved by the ethical committee for Laboratory Animals Care and Use of Dalian Medical University.

### 4.4. Xenograft Models

HeyA8 cells (5 × 10^5^/mL) suspended in 0.2 mL phosphate buffered saline (PBS) were injected in the right axilla of nude mice (16 males and 16 females). After 7 days, the ovarian tumor-bearing mice were randomly divided into four groups (*n* = 8): 0.9% normal saline group which was used as a control group, 0.075 mg/kg rLj-RGD3 group, 0.15 mg/kg rLj-RGD3 group and 0.3 mg/kg rLj-RGD3 group. All nude mice were intraperitoneally administered with normal saline or rLj-RGD3 every day for 4 weeks. And the body weight of the above nude mice was recorded every day.

### 4.5. Tumor Weight and Volume

The tumor volume was calculated based on the following formula: 1/2 × length × width^2^. During the four weeks of the study, the length and width of the tumors were monitored with Vernier calipers every 3 days. After 28 days, these HeyA8 xenografted mice were sacrificed and their tumors were excised. Subsequently, the weight and volume of these tumors were recorded and calculated accordingly. The inhibitory rate (IR) was calculated according to the following formula: IR = (1 − tumor weight/volume in the rLj-RGD3 treated groups/tumor weight/volume in the control group) × 100%.

### 4.6. H&E, Hoechst 33258 and TUNEL Staining

After normal saline or rLj-RGD3 administration for 28 days, the tumors of the xenogafted mice were excised for H&E staining, TUNEL assay, as well as western blot analysis. Firstly, the tumor tissues were fixed with 4% paraformaldehyde and embedded in paraffin. Secondly, the paraffin sections were placed at 60 °C for 1 h, and then placed into the dimethylbenzene for 10 min. Subsequently, the sections were respectively put into the 100%, 90%, 80%, 70%, 60% and 0% alcohol solution in sequence for 5 min. Thirdly, the above sections were stained with hematoxylin for 5 min and washed with ddH_2_O for 5 min, respectively. After washing with 3% hydrochloric acid in 95% ethyl alcohol and ddH_2_O for 30 s respectively, the sections were stained with eosin for 2 min. In the Hoechst 33258 staining assay, the sections were stained with Hoechst 33258 for 5 min. Furthermore, the sections were also stained with TUNEL assay kit (Roche, Shanghai, China). Finally, the sections were washed with PBS for three times, and then were dehydrated, mounted and observed by an inverted microscope (Nikon, Tokyo, Japan). The areas of fibrosis in H&E staining assay and the number of apoptotic cells were quantified by Image J. The fibrosis ratio in the normal saline group was defined as 1. The relative fibrosis ratio in the rLj-RGD3 groups was calculated with the formula: the areas of fibrosis in the rLj-RGD3 groups/the areas of fibrosis in the normal saline group. The positive rates of the apoptotic cells were calculated with the formula: the number of the cells with positive TUNEL staining/the number of the cells with positive Hoechst 33258 staining × 100%.

### 4.7. Western Blot

The tumors from the normal saline and rLj-RGD3 treated mice were grinded with a homogenizer at 4 °C in the cell lysis buffer containing phenylmethanesulfonyl fluoride (PMSF, Solarbio, Beijing, China). Then, the protein samples were prepared for 12% SDS-PAGE. The concentration of the above protein samples was measured with a bicinchoninic acid (BCA) kit. After SDS-PAGE, the proteins samples were transferred to the polyvinylidene difluoride (PVDF) membranes at 4 °C. Subsequently, the membranes were respectively blocked with 5% skim milk at room temperature for 1 h and then were respectively incubated with anti-Bcl-2 antibody (Cell Signaling Technology, Boston, MA, USA), anti-caspase 3 antibody (Cell Signaling Technology), anti-FAK antibody (Cell Signaling Technology), anti-p-FAK antibody (Cell Signaling Technology), anti-PI3K antibody (Cell Signaling Technology), anti-p-PI3K antibody (Cell Signaling Technology), anti-AKT antibody (Cell Signaling Technology), anti-p-AKT antibody (Cell Signaling Technology), and anti-β-actin antibody (Cell Signaling Technology) at a ratio of 1:1000 at 4 °C overnight. In order to remove the non-specific binding, the membranes were washed with phosphate buffered saline tween (PBST) buffer for three times. Next, the horseradish peroxidase (HRP)-IgG goat anti-rabbit (mouse) antibody was used to incubate with the above membranes at a ratio of 1:5000 at room temperature for 1–2 h. Finally, the membranes were washed with PBST and incubated with ECL kit. The blots were visualized with FluorChem Q (Proteinsimple, San Jose, CA, USA), and analyzed with Alpha View SA (Proteinsimple).

### 4.8. Survival Assays

In order to analyze whether rLj-RGD3 could affect the survival of nude mice bearing HeyA8 tumors, twenty eight nude mice (half male and female) were also injected with HeyA8 cells (5 × 10^5^/mL) in the right axilla. After 7 days, the above mice were administrated with normal saline and rLj-RGD3 in the manner which was similar to the procedure of 4.4. Finally, the survival days of the above mice were recorded. And the prolonged life span was calculated based on the formula: (the survival days in the rLj-RGD3 groups-the survival days in the normal saline group)/the survival days in the normal saline group × 100%.

### 4.9. Statistical Analysis

All the experiments were performed in triplicate. The data shown represent the mean ± SD. SPSS Statistics 22 (IBM, New York, NY, USA) was used to calculate the significant differences between the normal saline and rLj-RGD3 treated groups: * *p* < 0.05; ** *p* < 0.01 and *** *p* < 0.001.

## 5. Conclusions

In summary, rLj-RGD3 could effectively prolong the survival and inhibit the growth of tumors in nude mice bearing HeyA8 xenografts without affecting their body weight. Based on our microscopic observation and western blot analysis, rLj-RGD3 reduced the volume and weight of the excised tumors, probably through apoptosis and the suppression of the FAK/PI3K/AKT pathway. The above data suggested that rLj-RGD3 might be a safe drug to effectively treat ovarian cancer patients.

## Abbreviations

The following abbreviations are used in this manuscript:

AKT	Protein kinase B
BCA	Bicinchoninic acid
DMEM	Dulbecco’s modified Eagle medium
ECM	Extracellular matrix
FAK	Focal adhesion kinase
FBS	Fetal bovine serum
H&E	Hematoxylin and eosin
HRP	Horseradish peroxidase
IR	Inhibitory rate
*L. japonica*	*Lampetra japonica*
PBS	Phosphate buffered saline
PBST	Phosphate buffered saline tween
PI3K	Phosphoinositide 3-kinase
PMSF	Phenylmethanesulfonyl fluoride
PVDF	Polyvinylidene difluoride
RGD	Arg-Gly-Asp
SD	Sprague Dawley
SDS-PAGE	Sodium dodecyl sulfate-polyacrylamide gel electrophoresis
TME	Tumor microenvironment
